# Survivors of COVID-19 are at high risk of posttraumatic stress disorder

**DOI:** 10.1186/s41256-020-00155-2

**Published:** 2020-06-05

**Authors:** Shuiyuan Xiao, Dan Luo, Yang Xiao

**Affiliations:** Xiangya School of Public Health, Central South University, 238 Shang Mayuanling, Xiangya Road, Changsha City, 410078 Hunan China

**Keywords:** Coronavirus disease 2019, Posttraumatic stress disorder, Prevention

## Abstract

Posttraumatic stress disorder (PTSD) is a common mental disorder caused by major psychological trauma. It could result in serious distress and disability. Previous epidemic studies report high prevalence rates among people exposed to the trauma resulted from an infectious disease epidemic. While the control of the epidemic and care of patients with COVID-19 are still the dominant task of the whole world, this commentary calls for attention to early intervention and prevention of PTSD among huge numbers of COVID-19 survivors, their family members, health care professionals and other first-line helpers.

## Background

World Health Organization declared the 2019 coronavirus disease (COVID-19) outbreak a pandemic. As to May 11, 2020, 4, 006, 257 confirmed cases of COVID-19 and 278, 892 deaths have been reported to the World Health Organization (WHO), from more than 200 countries and territories [1]. It is still too early to predict how many people will be infected with the virus all over the world as the number of cases and deaths continue to rise. Meanwhile, millions of people are scared and even panic of the possible loss of health, life, and wealth. Experiencing or witnessing the suffering related to COVID-19 may result in high prevalence of posttraumatic disorder (PTSD), a mental disorder leading to serious distress and disability among survivors, family members, people who provide first aids and care (medical and public health professionals, police officers, etc.), and even among the general public. While control of the epidemic and care of patients with COVID-19 are still the dominant task of the whole world, this commentary calls for attention to early intervention and prevention of PTSD among affected populations.

PTSD is a common pathological outcome of a wide variety of traumatic events, from wars and disasters to individual events such as road traffic or work accidents [2]. Patients with PTSD live under the shadow of past trauma. Core symptoms of PTSD, as defined by the Diagnostic and Statistics of Mental Disorders, the fifth edition (DSM-5) [3] of the America Psychiatric Association, include persistent intrusion symptoms, persistent avoidance of stimuli, negative alterations in cognition or mood, and marked alterations in arousal and reactivity, all associated with the experienced traumatic event. PTSD results in clinically significant distress or impairment in social, occupational, or other important areas of functioning. Epidemiological data indicate that the median time for PTSD to remit is 36 months for individuals who sought help for any mental health problem (not necessarily for PTSD) and about 64 months for individuals who never sought help for a mental health problem. Approximately one-third of those who diagnosed with PTSD had a chronic course [4].

## Infectious disease epidemic and PTSD

Exposure to infectious disease epidemics results in a particular type of psychological trauma, which could be categorized into three groups. The first is directly experiencing and suffering from the symptoms and traumatic treatment. For example, dyspnea, respiratory failure, gatism, alteration of conscious states, threatening of death, tracheotomy, etc. are major trauma of patients with severe COVID-19. The second is witnessing of patients who suffer from, struggle against and die of the infectious disease, which has a direct impact on fellow patients, family members of patients, or people who directly provide aids and care for the patients. The third is experiencing the realistic or unrealistic fear of infection, social isolation, exclusion, and stigmatization. This directly affects patients, family members, care and help providers, or even the general public.

Epidemiological studies have demonstrated a rather high prevalence of mental health problems among survivors, victim families, medical professionals, and the general public after an epidemic of infectious disease, such as SARS, MERS, Ebola, flu, HIV/AIDS. While most of these mental health problems will fade out after the epidemic, symptoms of PTSD may last for a prolonged time and result in serious distress and disability. A systematic review of psychological consequences of infectious disease outbreak (after 2003 SARS outbreak, the H1N1 outbreak in 2009, and occupational exposure to HIV) indicates that the average prevalence of PTSD among health professionals was approximately 21% (ranging from 10 to 33%), and 40% of them reported persistently high PTSD symptoms 3 years after post exposure. PTSD symptoms were also significantly higher among exposed healthcare workers (HCWs) than unexposed control group, particularly among allied HCWs, followed by nurses and physicians [5]. A study of the long-term psychiatric morbidities among SARS survivors revealed that PTSD was the most prevalent long-term psychiatric condition. The cumulative proportion of patients with PTSD was 47.8%, while 25.5% continued to meet PTSD criteria at 30 months post-SARS [6]. Of the 116 people who survived from Ebola in Liberia, 76 (66%) met the DSM-IV diagnostic criteria of posttraumatic stress disorder 3 years after the outbreak (Nyanfor SS, Xiao SY: The Psychological Impact of the Ebola epidemic among Survivors in Liberia: a retrospective cohort study, submitted).

The feature and level of exposure to psychological trauma seems to be the most reliable predictor of PTSD after an infectious disease epidemic. Most epidemiological studies indicate the survivors reported highest prevalence of PTSD, followed by victim families, medical professionals providing care to patients with infectious diseases, and others. The female, the elderly, children, less educated, low-income groups are more vulnerable to PTSD, while comorbidity of chronic mental and somatic disorders, neurotic personality, lack of social connection and social support, etc. are possible risk factors, early psychosocial interventions are possible protective factors of PTSD [7].

## Prevention of PTSD after infectious disease epidemic

The importance of providing mental health service to people affected by the epidemic of infectious diseases are highly recognized by the academic society and the general public. In 2007, the Inter-Agency Standing Committee (IASC) announced *Guidelines on Mental Health and Psychosocial Support in Emergency Settings* [8], which has been widely adapted to direct mental health service after disasters, including infectious disease epidemics. The IASC guidelines are organized around a 4-tiered intervention pyramid: (1) restoring basic services and security for the affected population, (2) strengthening family and community networks, (3) providing distressed individuals with psychosocial support, and (4) providing specialized mental health intervention for severely affected survivors. Other strategies or models of intervention have also been practiced in various of settings [9]. However, systematic and well-designed intervention studies targeted at the prevention of PTSD after disasters are unavailable until now.

## Conclusion

With considerations of the already large and will still increasing number of people exposed to the current COVID-19, we believe it urgent to provide mental health service targeted at prevention of PTSD to survivors and other people exposed to COVID-19. Possible strategies include, but not limited to health education, psychosocial support and counselling service to the general population, as well as early intervention, including psychosocial support, psychotherapies, and pharmacological treatments to vulnerable and high-risk groups. Systematic and well-designed interventive trials with strict evaluation of the outcome, if possible, could also shed a light on the development of strategies and models of prevention of PTSD among people affected by epidemics of other infectious disease.

## Data Availability

Not applicable.

## Abbreviations

COVID-19: Coronavirus disease 2019
WHO: World Health Organization
PTSD: Posttraumatic disorder
DSM-5: Diagnostic and Statistics of Mental Disorders, the fifth edition
SARS: Severe Acute Respiratory Syndrome
MERS: Middle East Respiratory Syndrome
HIV: Human Immunodeficiency Virus
AIDS: Acquired immunodeficiency syndrome
HCWs: Healthcare workers
IASC: Inter-Agency Standing Committee
